# Targeting the deubiquitinase USP28 in cancer: navigating context-dependent mechanisms and therapeutic resistance

**DOI:** 10.3389/fimmu.2026.1844555

**Published:** 2026-07-23

**Authors:** Tongyong Luo, Shuncai Wu, Qingsong Wang, Jun Yin, Lijuan Zhang, Wenlong Yue, Xianmin Wang

**Affiliations:** 1 Pediatric Cardiology Center, Sichuan Provincial Women’s and Children’s Hospital/The Affiliated Women’s and Children’s Hospital of Chengdu Medical College, Chengdu, China; 2 Department of Ultrasound, West China Hospital Sichuan University Jintang Hospital, Jintang First People’s Hospital, Chengdu, China; 3 Department of Pediatrics, West China Hospital Sichuan University Jintang Hospital, Jintang First People’s Hospital, Chengdu, China; 4 Department of Surgery, West China Hospital Sichuan University Jintang Hospital, Jintang First People’s Hospital, Chengdu, China; 5 Department of Neurosurgery, West China Hospital Sichuan University Jintang Hospital, Jintang First People’s Hospital, Chengdu, China

**Keywords:** context-dependency, deubiquitinating enzyme, therapeutic target, tumorigenesis, USP28

## Abstract

Ubiquitin-specific protease 28 (USP28) is a deubiquitinating enzyme initially identified as a regulator that stabilizes p53 and c-MYC in response to DNA damage stress. Beyond its role in maintaining genomic integrity and cell cycle checkpoints, USP28 is implicated in diverse pathological processes. In various solid tumors, including lung, pancreatic, ovarian, and hepatocellular carcinomas, USP28 is markedly upregulated; it promotes proliferation, metabolic reprogramming, invasion, and therapeutic resistance by stabilizing oncoproteins such as c-Myc, STAT3, and SOX9. Conversely, in specific contexts like breast cancer and certain melanomas, USP28 deficiency drives malignant progression, revealing a context-dependent functional duality. These findings underscore the complexity of USP28 signaling and highlight its potential as a therapeutic target for precision medicine. This review summarizes the molecular characteristics and physiological functions of USP28, its context-dependent roles in neoplastic diseases, and its translational implications for targeted therapy and biomarker discovery.

## 1 The ubiquitin-proteasome system and deubiquitinases

The ubiquitin-proteasome system (UPS) mediates protein turnover in eukaryotes through a sequential enzymatic cascade. E1 ubiquitin-activating enzymes activate ubiquitin in an ATP-dependent manner and transfer it to E2 ubiquitin-conjugating enzymes. E3 ubiquitin ligases subsequently recognize specific substrates and catalyze ubiquitin transfer from E2 to the target, thereby conferring substrate specificity (1, 2). Deubiquitinases (DUBs) reverse this modification by cleaving ubiquitin-substrate bonds or editing ubiquitin chains, which regulates substrate stability and activity (3, 4).

Ubiquitin molecules can be linked via distinct lysine residues or the N-terminal methionine to form chains with distinct signaling outcomes. K48-linked chains generally direct substrates to the 26S proteasome for degradation (5, 6). K63-linked chains participate in non-degradative processes, including signal transduction and DNA repair (7, 8). K11-linked chains regulate cell-cycle progression, and K29-linked chains often cooperate with K48-linked chains to mediate proteasomal targeting (9, 10). These distinct chain topologies constitute a molecular code that determines substrate fate (11, 12). USP28 belongs to the ubiquitin-specific protease (USP) family of DUBs and recognizes K48-linked polyubiquitin chains on specific substrates to antagonize proteasomal degradation (3, 4).

## 2 Molecular characteristics and physiological functions of USP28

### 2.1 Molecular characteristics of USP28

**Figure 1:**
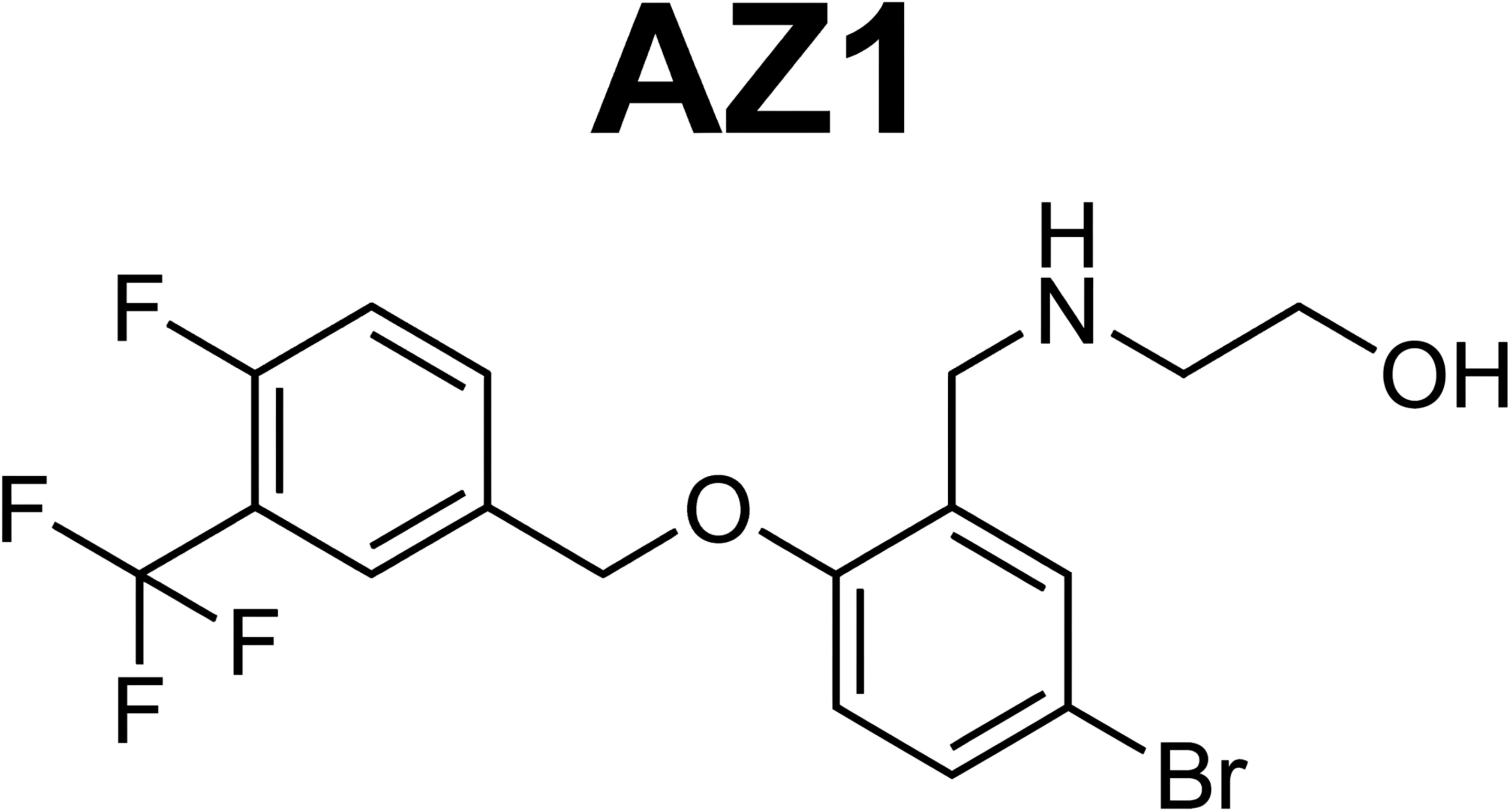
Chemical structure of the USP28 inhibitor AZ1. AZ1 is a heterobifunctional small-molecule inhibitor that targets the catalytic pocket of USP28. The structure contains a trifluoromethyl-substituted phenyl ring, an ether linkage, a bromophenyl moiety, and an ethanolamine side chain.

**Figure 2:**
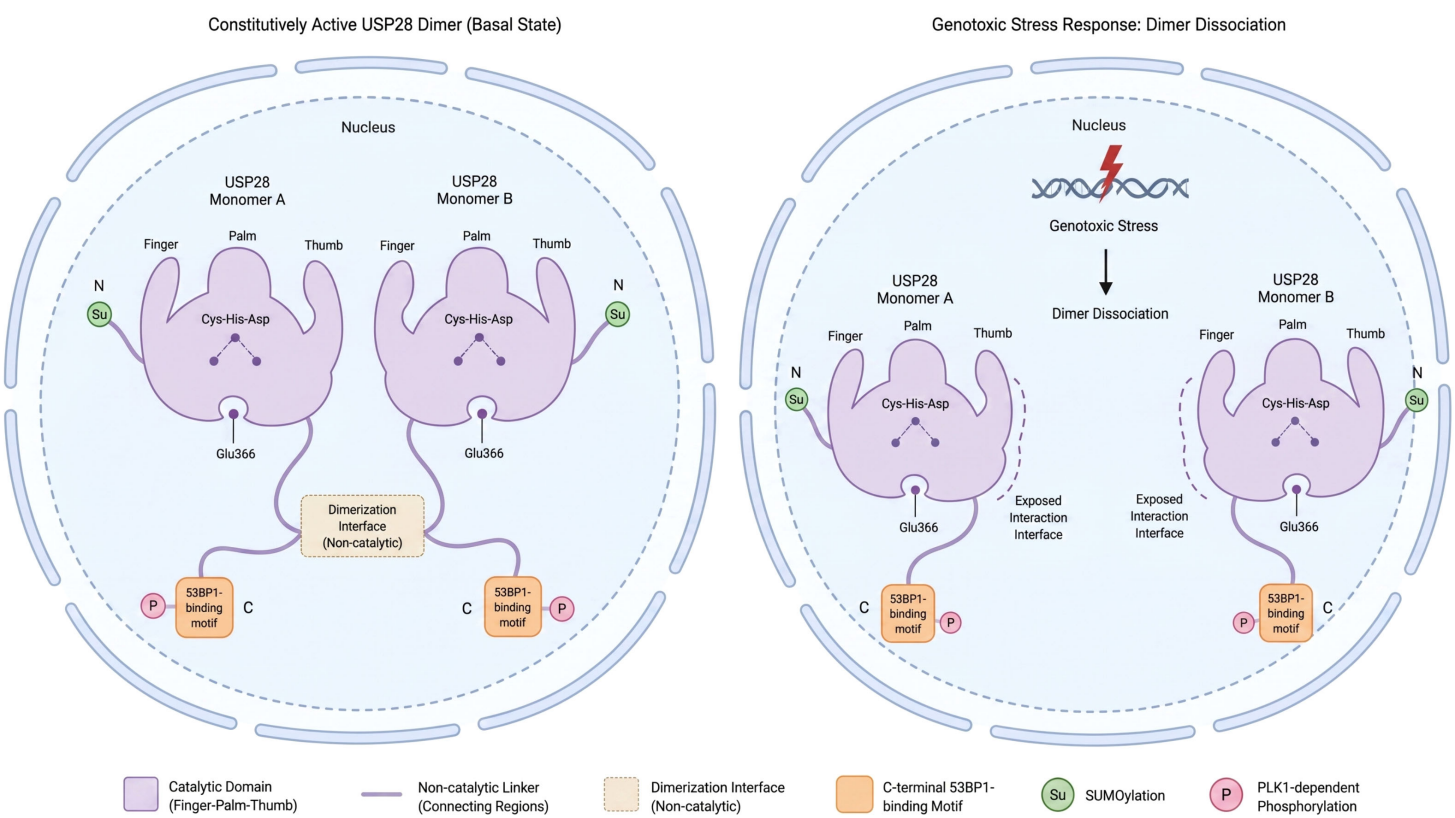
Structural domains and context-dependent activation mechanism of USP28. The schematic illustrates the essential structural motifs of USP28, including the catalytic triad (Cys-His-Asp) within the Finger-Palm-Thumb domains, and its key regulatory modifications (e.g., SUMOylation and PLK1-mediated phosphorylation). Under basal conditions, USP28 exists as an active dimer. In response to genotoxic stress, the dimer dissociates, exposing critical functional interfaces to interact with downstream substrates.

The USP28 (Ubiquitin Specific Peptidase 28) gene is located on human chromosome 1p34.2 and encodes a high-molecular-weight deubiquitinating enzyme belonging to the ubiquitin-specific protease (USP) family. Its amino acid sequence contains the canonical USP family conserved motifs, specifically the catalytic triad comprising cysteine (Cys), histidine (His), and aspartic acid (Asp) responsible for catalytic activity (13). High-resolution structural analysis reveals that the USP28 catalytic domain exhibits the classic fingers-palm-thumb folding pattern, yet its active site possesses unique conformational features. Notably, the glutamate residue at position 366 (Glu366) resides within a critical ligand-binding pocket. This residue is essential not only for maintaining pocket stability and basal enzymatic activity but also directly participates in the binding recognition of small-molecule inhibitors such as AZ1 and Vismodegib; mutation of this site impairs drug binding affinity, highlighting its dual role in catalytic function and drug sensitivity (14). The chemical structure of AZ1 is shown in **Figure 1**. Furthermore, the catalytic domain contains specific insertion sequences, which may provide a structural basis for substrate selectivity and allosteric regulatory motifs (15) (**Figure 2**).

The most distinctive molecular feature of USP28 is its existence as a constitutively active dimer, contrasting with the self-inhibitory tetramer formed by its homolog, USP25 (16). Structural biology evidence indicates that the USP28 dimerization interface relies on regions outside the catalytic domain, involving conserved hydrophobic clusters and charged residue networks. Moreover, its N-terminal region plays a pivotal role in regulating oligomeric equilibrium, determining the dynamic transition between dimeric and higher-order oligomeric states (15). This dimeric conformation is not static; rather, it directly regulates enzymatic activity through allosteric effects: the dimerized state can restrict its recruitment capacity for specific substrates such as the PAF1c complex, whereas genotoxic stress-induced dimer dissociation exposes new interaction interfaces, thereby activating downstream signaling pathways (17).

Beyond the catalytic core, the non-catalytic domains of USP28, particularly the C-terminal domain, play a decisive role in mediating specific protein-protein interactions. This region contains a specific 53BP1-binding motif, whose function strictly depends on PLK1 kinase-mediated phosphorylation. Clinical genomic data reveal that missense mutations frequently occurring in cancers are concentrated in this C-terminal binding domain rather than the catalytic domain. These mutations disrupt the interaction between USP28 and 53BP1, leading to the failure of the p53 stabilization mechanism and exacerbating genomic instability, underscoring the driving role of this non-catalytic region in tumorigenesis (18). Additionally, other functional motifs exist on the USP28 surface, such as the region for binding the E3 ligase DTX3L; together, they form an antagonistic feedback loop at DNA double-strand break sites, regulating the choice of repair pathways (19).

The molecular properties of USP28 are also dynamically regulated by various post-translational modifications, among which SUMOylation is particularly critical. Under normoxic conditions, USP28 is SUMOylated, exhibiting moderate deubiquitinating activity. Under hypoxic stress, the deSUMOylase SENP1 mediates the deSUMOylation of USP28, activating its enzymatic activity and promoting HIF-1α protein accumulation, thereby forming a SENP1-USP28-HIF-1α positive feedback loop (20). Furthermore, DYRK2 kinase can promote USP28 ubiquitination and degradation via phosphorylation, while USP28 conversely stabilizes DYRK2 protein levels, constituting a bidirectional regulatory feedback loop (21).

### 2.2 Physiological functions of USP28

**Figure 3:**
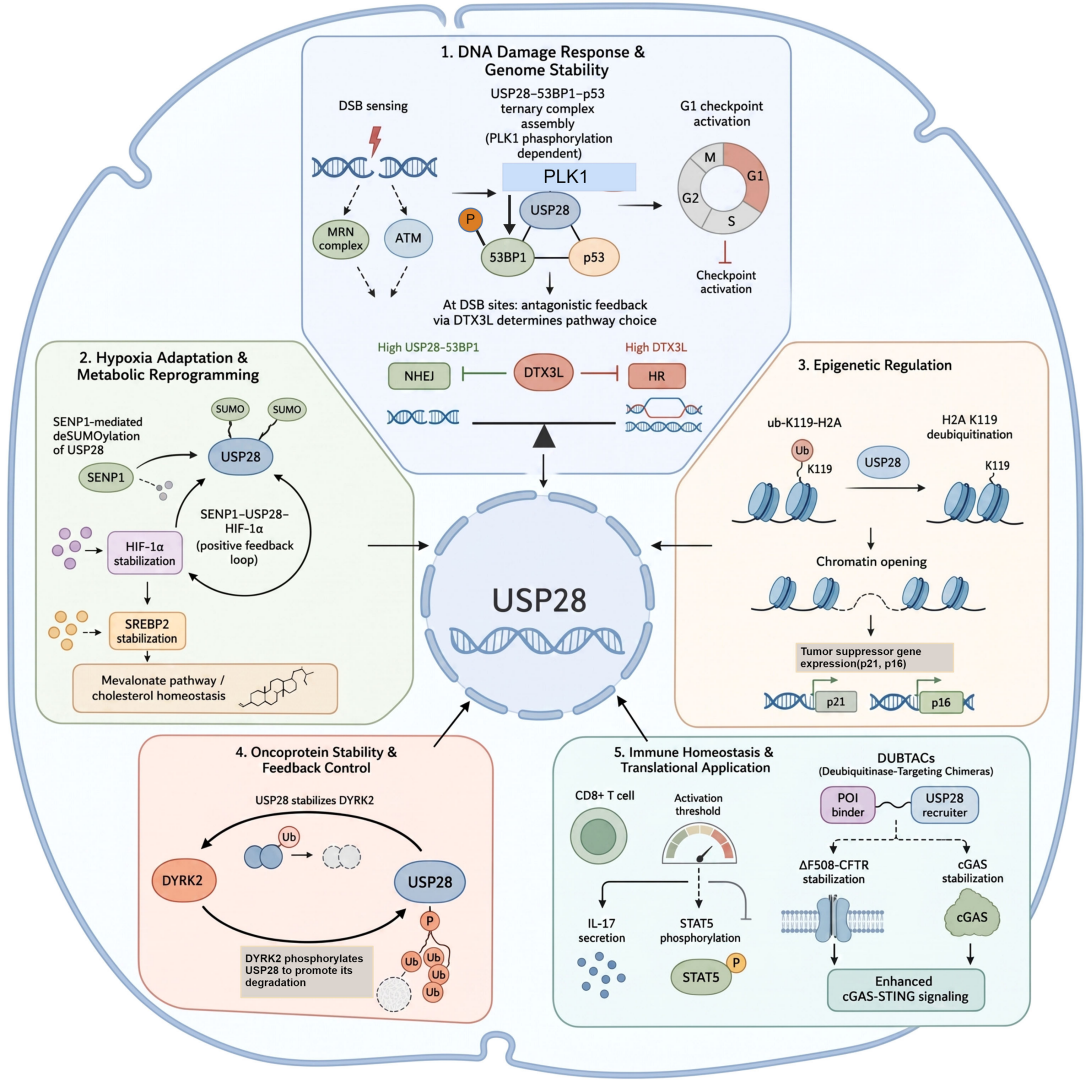
Physiological functions and molecular signaling networks of USP28. USP28 modulates diverse cellular processes, categorized into five key modules: (1) DNA damage response and genomic stability, highlighted by the competitive regulation of the 53BP1-p53 axis; (2) metabolic reprogramming and hypoxia adaptation (e.g., HIF-1α stabilization and SREBP2-mediated lipid synthesis); (3) epigenetic regulation of chromatin accessibility; (4) kinase-mediated feedback control (e.g., DYRK2-mediated phosphorylation and subsequent degradation of USP28);(5) immune homeostasis alongside emerging targeted protein degradation strategies (DUBTACs).

USP28 was initially identified as a key positive regulator of the p53 pathway, stabilizing the p53 protein via deubiquitination in response to DNA damage stress. Subsequent research has elucidated the functions of USP28 in non-canonical pathways, particularly in cell cycle surveillance, metabolic reprogramming, and epigenetic modification, indicating that it serves as a central hub connecting genomic stability, metabolic homeostasis, and environmental stress signals (22) (**Figure 3**).

#### 2.2.1 Regulation of DNA damage response and cell cycle checkpoints

As a core component of the DNA damage response (DDR) network, USP28 regulates the stability and localization of key effector proteins at multiple levels. Ionizing radiation, platinum-based chemotherapeutics such as cisplatin, topoisomerase inhibitors, ultraviolet light, and reactive oxygen species represent common sources of genotoxic stress. Mechanistically, USP28 forms a specific USP28-53BP1-p53 ternary complex with 53BP1 via its C-terminal domain. The assembly of this complex strictly depends on PLK1 kinase-mediated phosphorylation of 53BP1 (23). This ternary binding mode limits the access of E3 ubiquitin ligases such as MDM2 to p53 and protects p53 from degradation through steric hindrance, thereby triggering G1 phase cell cycle arrest (17). If the C-terminal binding motif of USP28 is mutated at frequent cancer mutation sites, its interaction with 53BP1 is weakened, preventing effective p53 accumulation under mitotic stress and compromising genomic integrity (24).

USP28 also regulates the choice of DNA double-strand break (DSB) repair pathways by antagonizing the E3 ligase DTX3L. At DSB sites, DTX3L mediates K48-linked ubiquitination of USP28 to promote its degradation, while USP28 conversely deubiquitinates and stabilizes both DTX3L and itself, forming a negative feedback loop (19). This dynamic balance determines the priority between non-homologous end joining (NHEJ) and homologous recombination (HR) pathways: high USP28 activity tends to promote NHEJ repair, whereas its deficiency may lead to a shift in repair pathway preference. Additionally, USP28 enhances the transmission efficiency of the ATM-Chk2-p53 signaling axis by stabilizing the CHK2 kinase, ensuring that cells can rapidly initiate checkpoint mechanisms upon exposure to ionizing radiation or replication stress (22, 25).

#### 2.2.2 Regulation of lipid metabolic reprogramming and cholesterol synthesis

USP28 plays a critical role in maintaining metabolic homeostasis, particularly by driving the mevalonate (MVA) pathway through direct regulation of Sterol Regulatory Element-Binding Protein 2 (SREBP2). USP28 specifically recognizes and binds to the mature form of SREBP2 (nSREBP2), removing its K48-linked polyubiquitin chains, thereby preventing its degradation by the proteasome (26, 27). This regulation exhibits high substrate specificity: USP28 does not affect the stability of SREBP1 or other metabolic transcription factors, but specifically maintains the transcriptional levels of intracellular cholesterol synthesis enzymes. In solid tumors, oxygen tension frequently falls below 2%, which stabilizes HIF-1α protein and reprograms cellular metabolism. Functional experiments confirm that USP28 deficiency leads to a significant shortening of the nSREBP2 half-life and downregulation of key enzymes such as HMGCR, markedly increasing the sensitivity of statin-sensitive tumors, such as squamous cell carcinoma, to cholesterol synthesis inhibitors (28).

#### 2.2.3 Differential regulation of specific substrates and tissue specificity

The regulatory effects of USP28 on different substrates vary depending on cell type and microenvironmental signals. In ovarian cancer, USP28 stabilizes the transcription factor SOX9, indirectly upregulating the expression of DNA repair genes such as SMARCA4, UIMC1, and SLX4, thereby conferring resistance to PARP inhibitors such as olaparib in cancer cells. Conversely, in breast cancer models, USP28 exhibits tumor-suppressive functions; its deficiency promotes epithelial-mesenchymal transition (EMT) and angiogenesis, an effect that is independent of the classical p53 or HIF-1α pathways (25, 29, 30). This context-dependent functional duality suggests that USP28 substrate selectivity may be regulated by tissue-specific cofactors or competitive binding proteins. For instance, in certain tissues, USP28 may form unique complexes with specific E3 ligases, thereby altering its substrate recognition profile (31–33).

#### 2.2.4 Role in epigenetic modification and chromatin remodeling

Beyond regulating protein stability, USP28 directly participates in epigenetic regulation at the level of histone modification. USP28 has been identified as a specific deubiquitinase for histone H2A lysine 119 (ub-K119-H2A). Under normal physiological conditions, USP28 maintains chromatin in an open state by removing ubiquitin marks from H2A, promoting the transcriptional expression of cell cycle inhibitors such as p21 and p16 (26). When USP28 function is lost, ub-K119-H2A levels increase, leading to chromatin compaction and silencing of key tumor suppressor genes, thereby accelerating cell proliferation (28, 34). This finding expands the functional scope of USP28 from protein homeostasis to chromatin remodeling, revealing a mechanism by which it maintains cellular homeostasis at the epigenetic level.

#### 2.2.5 Participation in immune homeostasis regulation and DUBTACs

USP28 is involved in T cell differentiation and inflammatory responses. USP28 deficiency results in increased CD8^+^ T cell numbers and impaired function, characterized by reduced IL-17 secretion and elevated STAT5 phosphorylation, indicating that USP28 participates in maintaining the activation threshold and effector function of T cells (35).

Deubiquitinase-targeting chimeras (DUBTACs) recruit DUBs to specific substrates to remove degradative ubiquitin chains. USP28-recruiting DUBTACs consist of a USP28-binding ligand, a chemical linker, and a target-protein recognition module (36–38). The heterobifunctional molecule positions USP28 near the substrate to cleave K48- or K63-linked polyubiquitin chains and block proteasomal degradation, thereby achieving targeted protein stabilization—a mechanism distinct from PROTAC-mediated degradation (36, 39). Two USP28-directed DUBTACs have been reported. A CFTR DUBTAC stabilizes the cystic fibrosis mutant ΔF508-CFTR and partially restores its membrane localization (36). A cGAS DUBTAC increases cGAS protein levels, which amplifies cGAS-STING signaling and enhances innate antitumor immunity (36). DUBTACs enable functional reprogramming rather than catalytic suppression and can stabilize tumor suppressors or restore the function of loss-of-function mutants (36, 39). Nevertheless, current designs depend on non-covalent USP28 ligands with modest affinity. The substrate scope remains limited to CFTR and cGAS; DUBTACs targeting classical tumor suppressors have not been developed with USP28 (40). The large molecular weight of heterobifunctional compounds restricts membrane permeability, and off-target deubiquitination remains a concern.In addition, efficacy data are currently limited to cell-based assays; animal pharmacokinetic and toxicity data are not yet available (36, 39).Furthermore, first-in-class PPARγ DUBTACs have been developed using USP28 as the recruiting DUB to target cancer metabolism. The lead compounds MS1727–MS1730 effectively stabilize PPARγ in both HeLa and MDA-MB-231 cells in a concentration- and time-dependent manner, requiring binding to both USP28 and PPARγ. These PPARγ DUBTACs significantly suppress cancer cell proliferation and upregulate PPARγ downstream target genes including CD36, FABP4, LPL, and FAS, providing a new potential anticancer therapeutic approach through metabolic reprogramming (36).

Collectively, USP28 regulates DNA repair, metabolic flux, hypoxic adaptation, and epigenetic status through ternary complex assembly, competitive antagonism, and post-translational modification switches. These mechanisms vary with cell type, substrate composition, and microenvironmental signals, and account for the role of USP28 in tumorigenesis, metabolic reprogramming, and immune regulation.

### 2.3 USP28 in tumor immunity and immunotherapy

#### 2.3.1 USP28 and the tumor immune microenvironment

Emerging evidence indicates that USP28 is a critical modulator of the tumor immune microenvironment. The dual USP25/28 inhibitor CT1113 triggers a robust cytotoxic CD8^+^ T cell-dependent antitumor immune response in multiple immunologically “cold” tumor models. Mechanistically, CT1113 reverses the immunosuppressive microenvironment by increasing CD8^+^ T cell infiltration and activity while attenuating M2-like tumor-promoting macrophage polarization, as confirmed by flow cytometry and single-cell RNA sequencing. At the molecular level, YTHDF2 is identified as a redundant substrate of USP25/28; dual inhibition promotes YTHDF2 degradation, thereby abolishing YTHDF2-mediated complement activation and immunosuppression. Pharmacological or genetic inhibition of USP25/28 significantly potentiates anti-PD-1 therapy in murine “cold” tumor models without obvious systemic toxicity (41).

#### 2.3.2 USP28 and cGAS-STING pathway activation

USP28-based deubiquitinase-targeting chimeras (DUBTACs) have been harnessed to activate innate antitumor immunity. USP28-recruiting cGAS DUBTACs (MS2099 and MS2100) effectively stabilize cGAS protein, elevate cGAMP levels, and promote downstream STING phosphorylation and IRF3 activation, thereby amplifying the cGAS-STING signaling pathway and eliciting antiproliferative effects in cancer cells (36). This approach expands the therapeutic application of USP28 modulation from catalytic inhibition to substrate-specific stabilization for immune modulation.

#### 2.3.3 USP28 and T-cell homeostasis

USP28 is also involved in T cell differentiation and inflammatory responses. USP28 deficiency results in increased CD8^+^ T cell numbers but impaired function, characterized by reduced IL-17 secretion and elevated STAT5 phosphorylation, indicating that USP28 participates in maintaining the activation threshold and effector function of T cells (35). These findings suggest that USP28 activity fine-tunes adaptive immunity beyond its canonical roles in DNA repair and oncogenesis.

## 3 Roles and mechanisms of USP28 in human cancers

**Figure 4:**
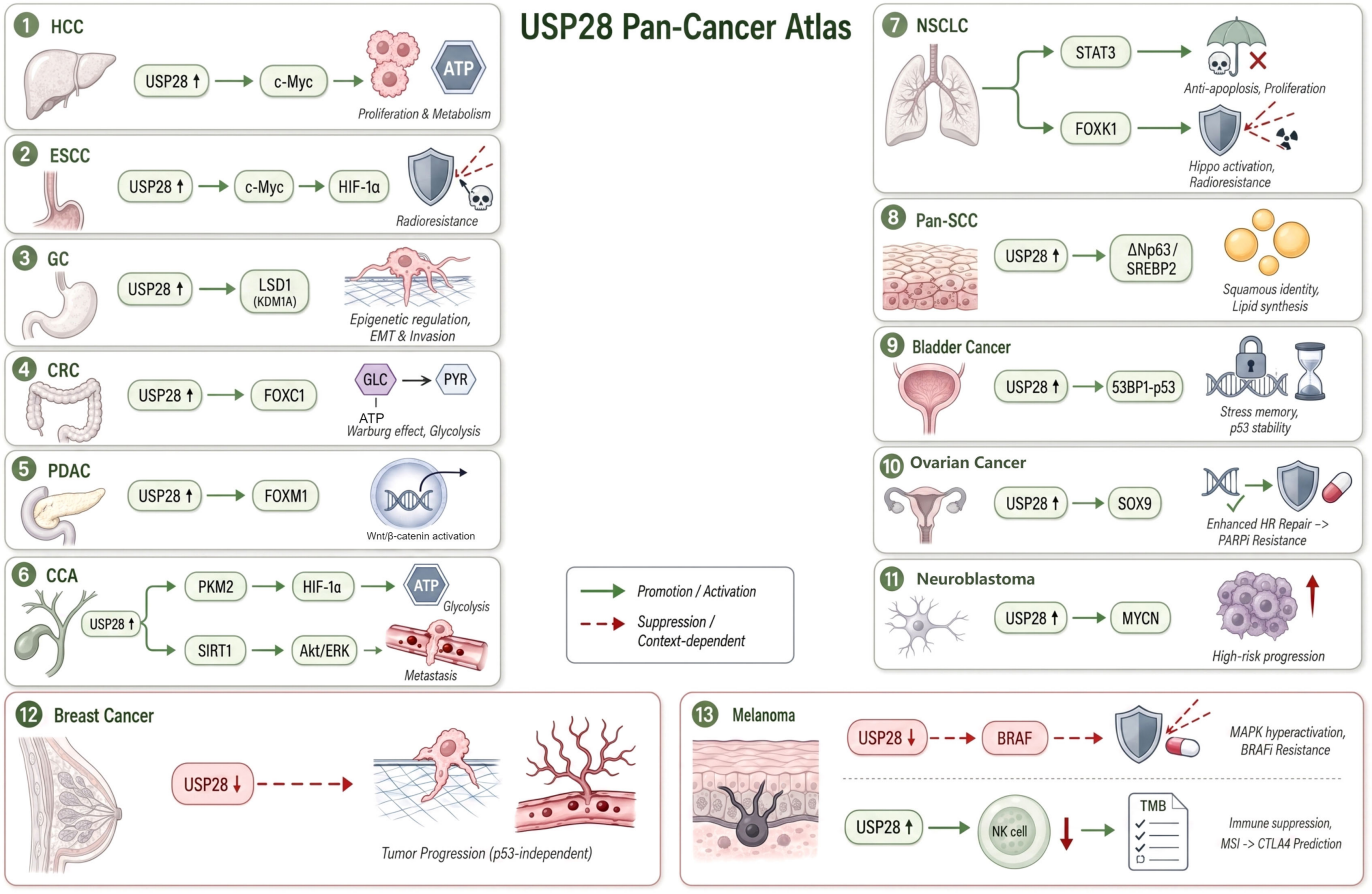
Context-dependent oncogenic and resistance mechanisms of USP28 across human cancers. This disease-centric diagram summarizes the highly heterogeneous roles of USP28 in pan-cancer progression. The upper and lateral panels delineate canonical gain-of-function mechanisms, where USP28 overexpression stabilizes distinct oncogenic substrates (green solid arrows) to drive tumorigenesis and therapy resistance in various malignancies. Conversely, the bottom panels highlight context-dependent dual roles, featuring loss-of-function-driven tumor progression and targeted therapy resistance (red dashed arrows) in breast cancer and BRAF-mutant melanoma, as well as the USP28-induced immunosuppressive microenvironment (characterized by reduced NK cell infiltration) in specific melanoma subsets.

USP28 has emerged as a key regulator in diverse pathological processes with context dependency. Although it is upregulated and promotes tumorigenesis in many solid tumors, it also exerts tumor-suppressive effects in some cancers. USP28 directly deubiquitinates and stabilizes c-MYC via K48-linked polyubiquitin chain removal in multiple solid tumors. The context-dependent roles and molecular mechanisms of USP28 across major human malignancies are summarized in **Table 1**. This section summarizes the distinct mechanisms by which USP28 participates in neoplastic diseases (**Figure 4**).

**Table 1:** Context-Dependent Roles and Molecular Mechanisms of USP28 in Neoplastic Diseases.

Disease type	USP28 expression	Key substrate(s)	Downstream pathway(s)/mechanism	Key experimental evidence	Reference
Hepatocellular carcinoma (HCC)	Markedly upregulated	c-Myc, KRT1, TCF7L2	miR-216b/USP28/c-Myc/Cyclin E; USP28/KRT1/IFITM3; USP28/TCF7L2/Wnt-β-catenin	Upregulation correlates with poor prognosis and sorafenib resistance; knockdown inhibits tumor growth	(42) (43) (44)
Non-small cell lung cancer (NSCLC)	Abnormally upregulated	STAT3, FOXK1, SIRT1	STAT3 signaling; Hippo/FOXK1 pathway; USP28/SIRT1 axis drives osimertinib resistance and glycolysis	Upregulation correlates with poor OS; knockdown induces apoptosis; USP28 depletion restores osimertinib sensitivity	(45) (46) (47) (48)
Pancreatic cancer	Markedly upregulated	FOXM1	USP28/FOXM1/Wnt-β-catenin positive feedback loop	Upregulation correlates with malignant phenotype and shortened survival	(49)
Cholangiocarcinoma (CCA)	Significantly upregulated	PKM2, SIRT1	USP28/PKM2/HIF-1α glycolysis; USP28/SIRT1/Akt/ERK	Upregulation correlates with poor prognosis; deletion inhibits migration and EMT	(50) (51)
Esophageal squamous cell carcinoma (ESCC)	Upregulated	c-Myc	c-Myc/HIF-1α inhibits DDR pathway, enhancing radioresistance	USP28 knockdown potentiates radiosensitivity	(52)
Colorectal cancer (CRC)	Markedly upregulated	FOXC1	USP28/FOXC1/glycolysis genes; promotes Warburg effect	Inhibition induces FOXC1 degradation and suppresses metabolic reprogramming	(53)
Ovarian cancer	Upregulated	SOX9	USP28/SOX9/DNA repair genes (SMARCA4 etc.); enhances HR repair; mediates PARP inhibitor resistance	Upregulation correlates with poor prognosis and olaparib resistance; AZ1 restores olaparib sensitivity	(25) (54)
Squamous cell carcinoma (SCC, pan-site)	Universally upregulated	ΔNp63, c-MYC, SREBP2	Maintains malignant phenotype via oncoprotein stabilization; SREBP2/mevalonate pathway drives cholesterol synthesis	Inactivation induces oncoprotein degradation and tumor regression; USP28 deletion enhances statin sensitivity	(26) (27) (28)
Gastric cancer	Significantly upregulated	LSD1/KDM1A	Epigenetic regulation via LSD1; S-phase cell cycle regulation	Upregulation correlates with reduced differentiation and enhanced metastasis	(55)
Bladder cancer	Markedly upregulated	p53 (indirect via 53BP1)	PLK1-dependent binding to 53BP1; maintains mitotic stress memory and p53 stability	Upregulation correlates with p53 expression and tumor progression	(56)
Neuroblastoma	Upregulated	MYCN	Direct deubiquitination stabilizes MYCN; prolongs half-life	Correlates with MYCN amplification and poor prognosis; knockdown reduces MYCN stability	(57)
Melanoma	Heterogeneous (upregulated in subsets, deleted in others)	BRAF (when deleted)	Upregulation: correlates with immune microenvironment (NK cells), TMB, MSI; predicts CTLA-4 response. Deletion: stabilizes BRAF, activates MAPK, mediates BRAF inhibitor resistance	Upregulation predicts CTLA-4 inhibitor response; deletion reduces BRAF inhibitor sensitivity	(58)
Breast cancer	Functional heterogeneity	c-MYC (oncogenic); unknown (tumor-suppressive)	Oncogenic: stabilizes nuclear oncoproteins. Tumor-suppressive: deletion promotes EMT, proliferation and angiogenesis (p53/HIF-1α-independent)	Upregulation in invasive ductal carcinoma correlates with improved survival	(38) (59)
Chronic lymphocytic leukemia (CLL)	Deleted in ~90% of del(11q) patients	NICD (NOTCH1 intracellular domain)	USP28 interacts with NICD independently of FBXW7 and PEST domain; stabilizes NICD and enhances NOTCH1 signaling; 15 target genes dysregulated (IRF8, SOX5, KANK1, TOX2, ZFHX3, APH1B, etc.)	AZ1 suppresses NOTCH1 activation in primary CLL; AZ1+venetoclax synergistically reduces viability in high-NOTCH1 samples	(60)

### 3.1 Hepatocellular carcinoma

Hepatocellular carcinoma (HCC) is a highly lethal malignancy globally. Due to its insidious early symptoms, most patients are diagnosed at advanced stages, losing the opportunity for surgical resection (61). Although targeted therapy and immunotherapy have made some progress, tumor heterogeneity and drug resistance remain the primary causes of poor prognosis, necessitating the integration of multi-omics data to promote more precise molecular subtyping and personalized treatment strategies (62). USP28 is a key oncogene involved in HCC progression. Its expression is significantly elevated in HCC tissues, and high expression is closely associated with poor prognosis and shorter overall survival in patients. Functional studies show that high USP28 expression promotes the proliferation, migration, and invasion of liver cancer cells and is directly correlated with resistance to the targeted drug sorafenib; knocking down USP28 effectively inhibits tumor growth and enhances drug sensitivity (42). Sorafenib inhibits multiple kinases including RAF and VEGFR, thereby blocking tumor proliferation and angiogenesis.

USP28 drives HCC progression through multiple signaling axes. First, miR-216b is downregulated in HCC, relieving its targeted inhibition of USP28, which subsequently leads to upregulation of the USP28 substrate c-Myc and downstream Cyclin E, accelerating cell cycle progression (43). Second, USP28 directly interacts with Keratin 1 (KRT1), stabilizing the KRT1 protein via deubiquitination, which in turn promotes the expression of Interferon-Induced Transmembrane Protein 3 (IFITM3), ultimately driving tumor invasion and metastasis (42). Additionally, USP28 can activate the Wnt/β-catenin signaling pathway by stabilizing TCF7L2, further amplifying pro-carcinogenic effects (44). In conclusion, USP28 promotes HCC progression through multiple pathways including miR-216b/USP28/c-Myc, USP28/KRT1/IFITM3, and USP28/TCF7L2/Wnt, making it a potential prognostic biomarker and therapeutic target. In HCC, USP28 converges on the Wnt/β-catenin pathway through TCF7L2 stabilization while simultaneously engaging the KRT1/IFITM3 axis for metastasis—a dual mechanism not observed in other cancer types.

### 3.2 Non-small cell lung cancer

Lung cancer has long ranked first globally in both incidence and mortality rates, with Non-Small Cell Lung Cancer (NSCLC) accounting for the highest proportion (45, 63). Although targeted therapies such as EGFR-TKIs have achieved breakthroughs, the emergence of acquired resistance and radio-resistance remains an urgent clinical challenge, severely limiting patient survival benefits (64, 65). USP28 is abnormally overexpressed in NSCLC, and its levels are significantly correlated with poorer survival prognosis. Functional experiments demonstrate that USP28 overexpression promotes the *in vitro* proliferation and *in vivo* xenograft growth of NSCLC cells, whereas knocking down USP28 induces tumor cell apoptosis and inhibits tumor progression (46).

USP28 maintains the survival advantage of tumors by regulating key signaling pathways. On one hand, USP28 directly binds to Signal Transducer and Activator of Transcription 3 (STAT3), enhancing STAT3 protein stability via deubiquitination, thereby continuously activating the STAT3 signaling pathway to drive unlimited cell proliferation (47). On the other hand, USP28 stabilizes the transcription factor FOXK1, subsequently activating the Hippo signaling pathway; this process not only promotes cell proliferation but also induces radio-resistance in tumor cells (48). Additionally, USP28 participates in regulating ubiquitination processes and DNA damage responses, maintaining cell cycle stability under genotoxic stress (66). Osimertinib is a third-generation EGFR tyrosine kinase inhibitor that targets EGFR-activating mutations and the T790M resistance mutation. Cisplatin is a platinum-based chemotherapeutic that induces DNA interstrand crosslinks and triggers apoptosis.

Notably, in extensive Squamous Cell Carcinoma (SCC), USP28 exhibits a lineage-dependent pro-carcinogenic mechanism. SCC typically possesses high genomic instability and relies heavily on the sustained expression of specific oncogenes to maintain its cell identity (45, 67). In this microenvironment, USP28 acts as a stabilizer of the core oncogenic transcription network: it promotes cell cycle progression and survival by deubiquitinating and stabilizing proteins such as ΔNp63 (a core factor maintaining tumor cell transcriptional identity and epithelial characteristics), c-MYC, c-JUN, and NOTCH1 (68). Genetic inactivation of USP28 or the use of low nanomolar concentration small-molecule inhibitors can disrupt the aforementioned oncogenic protein network, deprive tumor cells of their identity, and induce apoptosis (27). As one of the most common types of SCC—Lung Squamous Cell Carcinoma (LUSC)—USP28 is deeply involved in tumor metabolic remodeling. USP28 can activate the mevalonate (MVA)-mediated cholesterol and isoprenoid synthesis pathway by deubiquitinating and stabilizing SREBP2, providing metabolic support for rapid tumor cell growth; moreover, USP28 deficiency can significantly enhance the sensitivity of squamous cancer cells to statins (26). Statins inhibit HMG-CoA reductase and block the mevalonate pathway of cholesterol synthesis. This mechanism targeting cell identity and lipid metabolic reprogramming is not only present in LUSC but also has broad universality in SCCs of other sites, such as the head and neck (28).

Notably, recent evidence has uncovered an additional mechanism of USP28-mediated therapeutic resistance in lung adenocarcinoma. USP28 and its substrate SIRT1 are significantly upregulated in osimertinib-resistant H1975 cells (H1975/OSI) and osimertinib-resistant NSCLC tissues. USP28 directly interacts with and deubiquitinates SIRT1, thereby stabilizing SIRT1 protein and extending its half-life. Functionally, USP28 overexpression enhances cell proliferation and glycolysis (upregulating GLUT1 and HK2) while suppressing apoptosis, thereby conferring osimertinib resistance; conversely, USP28 depletion or SIRT1 knockdown restores osimertinib sensitivity in H1975/OSI cells both *in vitro* and *in vivo*. These findings identify the USP28/SIRT1 axis as a novel therapeutic vulnerability for overcoming third-generation EGFR-TKI resistance in EGFR-mutant NSCLC (45).

In summary, USP28 plays a core driving role in NSCLC and its squamous cell carcinoma subtype: at the overall NSCLC level, USP28 primarily drives tumor cell proliferation and chemo-radio-resistance through the STAT3 and Hippo/FOXK1 pathways; at the pan-SCC level (including LUSC), it further maintains tumor cell identity by stabilizing lineage transcription factors like ΔNp63 and reshapes the lipid metabolic network via the SREBP2 axis. Therefore, targeting USP28 combined with cisplatin or radiotherapy, or adopting a USP28 inhibitor plus statin metabolic intervention strategy for the squamous cell carcinoma subtype, may serve as a combination strategy to overcome treatment bottlenecks in NSCLC and various SCCs.

### 3.3 Pancreatic cancer

Pancreatic cancer is characterized by high aggressiveness and early metastasis, resulting in poor clinical prognosis (68). As one of the most malignant tumors, its high mortality rate makes it a leading cause of cancer-related deaths globally (69). Due to the lack of early specific diagnostic markers, approximately 80% of patients have distant metastases at diagnosis, rendering them ineligible for radical surgery, and they are generally resistant to existing chemoradiotherapy regimens, with a five-year survival rate of less than 10% (70). USP28 is a critical driver promoting the malignant progression of pancreatic cancer. Its expression level in pancreatic cancer tissues is significantly higher than in normal pancreas, and high expression is closely related to malignant phenotypes and shortened patient survival. Functional validation indicates that USP28 overexpression promotes pancreatic cancer cell proliferation, whereas its knockdown effectively inhibits tumor growth both *in vitro* and *in vivo* (13).

The core function of USP28 lies in stabilizing the transcription factor Forkhead Box M1 (FOXM1). USP28 prevents FOXM1 degradation by the proteasome through deubiquitination, thereby enhancing the activity of the Wnt/β-catenin signaling pathway and forming a USP28-FOXM1-Wnt/β-catenin positive feedback loop that drives tumor cell proliferation and inhibits apoptosis (49). Rescue experiments confirmed that restoring FOXM1 expression could reverse the anti-tumor effects brought about by USP28 silencing, confirming the dependency on this axis. In conclusion, USP28 drives pancreatic cancer progression by stabilizing FOXM1 to activate the Wnt pathway, serving not only as a potential diagnostic marker but also as a target to address treatment limitations. The USP28-FOXM1-Wnt/β-catenin positive feedback loop represents a lineage-specific dependency not shared by other gastrointestinal malignancies.

### 3.4 Cholangiocarcinoma

Malignant tumors of the biliary tract (including intrahepatic cholangiocarcinoma) are highly challenging to treat due to their strong invasiveness and high rate of early metastasis (71, 72). Approximately 60%–80% of patients present with vascular invasion or distant metastasis at diagnosis, missing the window for surgical intervention, and exhibit significant resistance to chemoradiotherapy, with a median survival of less than 6 months for advanced patients (73, 74). USP28 expression levels in cholangiocarcinoma (CCA) are significantly higher than in normal bile duct tissues, and high expression is closely associated with malignant phenotypes and poor prognosis. *In vitro* and *in vivo* experiments confirm that USP28 deficiency significantly inhibits the growth, migration, and epithelial-mesenchymal transition (EMT) processes of cholangiocarcinoma cells, suggesting its key role in driving tumor progression 13].

USP28 primarily functions by reshaping tumor metabolism and activating survival signaling pathways. First, USP28 enhances the stability of Pyruvate Kinase M2 (PKM2) through deubiquitination, subsequently activating the Hypoxia-Inducible Factor 1α (HIF-1α) signaling pathway, promoting glycolysis and energy supply to provide metabolic support for rapid tumor growth (50). Second, in intrahepatic cholangiocarcinoma, USP28 can also activate the Akt/ERK signaling pathway by deubiquitinating and stabilizing the SIRT1 protein, further promoting cell proliferation and metastasis (51). Animal models show that USP28 overexpression promotes tumor growth and lung metastasis, while SIRT1 overexpression can reverse the functional defects caused by USP28 deficiency. In summary, USP28 drives cholangiocarcinoma progression through multiple signaling pathways such as PKM2-HIF-1α and SIRT1-Akt/ERK, and may have therapeutic relevance. USP28-driven metabolic reprogramming in CCA uniquely couples glycolysis (via PKM2/HIF-1α) with Akt/ERK survival signaling, distinguishing it from the SREBP2-dependent lipid synthesis seen in SCC.

### 3.5 Esophageal squamous cell carcinoma

Esophageal squamous cell carcinoma (ESCC) is a common malignant tumor of the digestive tract, particularly prevalent in Asia (75–77). Its main clinical challenges include radio-resistance and early metastasis, leading to poor treatment outcomes and high recurrence rates, necessitating the search for sensitizing targets to improve patient prognosis (78–80). USP28 is a key oncogene in ESCC, and its expression level is significantly correlated with tumor stage and radio-resistance. Functional experiments show that knocking down USP28 significantly enhances the sensitivity of esophageal cancer cells to radiotherapy, suggesting that targeting USP28 may be an effective strategy to overcome radio-resistance in esophageal cancer (18). Cisplatin is a platinum-based chemotherapeutic that induces DNA interstrand crosslinks and triggers apoptosis.

USP28 promotes the accumulation of HIF-1α by stabilizing the oncoprotein c-Myc, thereby inhibiting the DNA damage response pathway and allowing tumor cells to survive under radiation exposure. This process involves a c-Myc-mediated downstream signaling cascade, ultimately leading to enhanced tumor cell tolerance to radiotherapy (43, 52). In conclusion, USP28 enhances radio-resistance in esophageal cancer by stabilizing c-Myc, representing a potential sensitizing therapeutic target. The c-Myc/HIF-1α axis in ESCC highlights a tissue-specific adaptation where USP28-mediated radioresistance is tightly linked to hypoxic microenvironmental pressure.

### 3.6 Colorectal cancer

Colorectal cancer (CRC) is the third most common malignant tumor globally. Although widespread screening has improved early diagnosis rates, the treatment of advanced metastatic colorectal cancer still faces challenges, particularly the frequent occurrence of resistance to targets such as EGFR. Moreover, tumor metabolic reprogramming (e.g., the Warburg effect) is a significant characteristic (81–85). USP28 expression is significantly elevated in colorectal cancer tissues, and its high expression is closely related to malignant tumor progression. Inhibiting USP28 induces the degradation of key transcription factors, significantly suppressing tumor cell proliferation and metabolic reprogramming, suggesting that the USP28-FOXC1 axis is a potential therapeutic target. USP28 stabilizes the transcription factor FOXC1 protein via deubiquitination, preventing its degradation by the proteasome. Stabilized FOXC1 subsequently upregulates the expression of glycolysis-related genes, promoting aerobic glycolysis (Warburg effect) and rapid proliferation of tumor cells (53). This finding reveals the role of USP28 in regulating energy metabolism and proliferation in colorectal cancer, providing a molecular basis for developing USP28-targeted therapeutic strategies. USP28-directed Warburg effect in CRC is notable for its reliance on FOXC1 rather than HIF-1α, suggesting distinct metabolic wiring compared to other USP28-driven tumors.

### 3.7 Ovarian cancer

The main clinical challenge for ovarian cancer patients is the development of resistance to chemotherapy drugs, particularly PARP inhibitors such as olaparib, leading to treatment failure and poor prognosis (25). Although initial treatment responses are often good, recurrence and resistance are almost inevitable outcomes for advanced ovarian cancer, necessitating the elucidation of resistance mechanisms to develop combination therapy strategies (30). USP28 is highly expressed in ovarian cancer tissues, and its levels are regulated by the β-catenin/YAP1/TBX5 complex-mediated transcription, showing a significant correlation with poor patient prognosis. Functional studies indicate that high USP28 expression is a key factor causing resistance to PARP inhibitors in ovarian cancer cells, while the use of the specific USP28 inhibitor AZ1 can restore tumor cell sensitivity to olaparib (54). Olaparib is a PARP inhibitor that induces synthetic lethality in tumors with homologous recombination deficiency.

USP28 stabilizes SOX9 protein levels by directly interacting with SOX9 and inhibiting FBXW7-mediated ubiquitination and degradation of SOX9. Stabilized SOX9 then binds to the promoters of DNA damage repair genes such as SMARCA4, UIMC1, and SLX4, enhancing homologous recombination repair capacity, ultimately leading to tumor resistance to PARP inhibitors (25). Knocking down USP28 or using inhibitors promotes SOX9 degradation, weakens DNA repair capacity, and thereby reverses the resistant phenotype (54). In summary, USP28 mediates ovarian cancer resistance to PARP inhibitors by stabilizing SOX9 to enhance DNA repair capacity, serving as a combination therapeutic target to overcome resistance. The USP28-SOX9 axis in ovarian cancer uniquely links DNA repair capacity to PARP inhibitor resistance, a mechanism less prominent in USP28-driven solid tumors outside the reproductive tract.

### 3.8 Gastric cancer

Gastric cancer is the fifth most common cancer globally and the third leading cause of cancer death, imposing a heavy burden especially in East Asia (86–88). Helicobacter pylori infection is a key etiological factor, and its eradication can significantly reduce incidence; other risks include high-salt diets, pickled foods, smoking, and EBV infection (55). Early diagnosis relies on staging, grading, and genetic testing, while treatment encompasses surgery, chemoradiotherapy, targeted therapy, and immunotherapy (89, 90). Patients with advanced gastric cancer often have poor prognosis due to peritoneal metastasis and chemotherapy resistance, with a five-year survival rate of less than 30%, urgently requiring the search for new prognostic markers and therapeutic targets (91, 92). USP28 expression is significantly upregulated in gastric cancer tissues, and its expression level is closely correlated with reduced tumor differentiation and enhanced metastatic ability. *In vitro* experiments indicate that inhibiting USP28 significantly reduces the proliferation and invasive capabilities of gastric cancer cells, suggesting its pro-carcinogenic role in gastric cancer progression (15). The pro-carcinogenic effect of USP28 may involve epigenetic regulatory pathways. Inhibiting USP28 can downregulate the expression of Lysine-Specific Demethylase 1 (LSD1/KDM1A), thereby suppressing the proliferation, invasion, and epithelial-mesenchymal transition (EMT) processes of gastric cancer cells (55); other studies point out that USP28 participates in S-phase cell cycle regulation in gastric cancer (66). In summary, USP28 promotes gastric cancer progression by regulating epigenetic factors such as LSD1, representing a potential diagnostic and therapeutic target. USP28-mediated epigenetic regulation via LSD1 in gastric cancer underscores its context-dependent engagement of chromatin modifiers rather than classical oncogenic transcription factors.

### 3.9 Bladder cancer

Urothelial carcinoma (bladder cancer) is the most common malignant tumor of the urinary system, characterized by high recurrence rates and progression risk (93). Although BCG instillation and chemotherapy are standard treatments, survival rates for patients with muscle-invasive bladder cancer and metastatic disease remain low, urgently requiring new prognostic markers to guide clinical decision-making (94, 95). USP28 expression in bladder cancer tissues is significantly higher than in normal tissues, and its high expression is positively correlated with p53 protein expression, closely related to the degree of tumor progression and patient prognosis (13). USP28 can mediate PLK1-dependent binding to 53BP1 via its C-terminal domain, which is crucial for maintaining mitotic stress memory and p53 stability; this may partially explain the mechanism of its co-expression with p53 and promotion of tumor progression in bladder cancer (56). In summary, USP28 is highly expressed in bladder cancer and acts synergistically with p53, serving as a potential indicator for predicting tumor progression and patient prognosis. The co-expression of USP28 with p53 in bladder cancer reflects a unique urothelial dependency on the USP28-53BP1-p53 axis for mitotic stress memory.

### 3.10 Neuroblastoma

Neuroblastoma is the most common cancer in infants and the most common extracranial solid tumor in children. Patients with high-risk disease have poor prognosis, accounting for 10% of pediatric cancer deaths (96). Among these, MYCN amplification is a major adverse prognostic factor, and such tumors are often resistant to conventional chemotherapy. Identifying key regulatory factors that can stabilize the MYCN protein is crucial for developing targeted therapies (97, 98). USP28 directly stabilizes the MYCN protein via deubiquitination, extending its half-life and thereby driving tumor cell proliferation (13). This mechanism exists in both MYCN-amplified and non-amplified tumors, indicating that USP28 is a universal driver across molecular subtypes. Functional validation shows that genetic knockdown or pharmacological inhibition of USP28 significantly reduces MYCN (and c-MYC) protein stability, effectively inhibiting the growth of high-risk neuroblastoma cells (57). Given the pivotal role of MYCN in the occurrence and development of neuroblastoma, USP28, as its upstream stabilizer, is considered a broad-spectrum therapeutic target for this disease, especially suitable for high-risk patient groups currently lacking effective targeted means. USP28-driven MYCN stabilization operates across both MYCN-amplified and non-amplified neuroblastoma subtypes, indicating a universal lineage dependency in pediatric neural crest-derived malignancies.

### 3.11 Melanoma

The global burden of malignant melanoma continues to rise, with prevalence, incidence, and DALYs increasing from 1990 to 2021, showing regional differences, and is expected to worsen over the next 15 years (99). Ocular melanoma accounts for about 5% of all melanomas; posterior uveal types often lack typical mutations, while iris and conjunctival types harbor mutations, with genetic characteristics influencing clinical decisions (100). The disease is prone to early metastasis, with brain metastasis being a major cause of patient death. Although the incidence of melanoma globally continues to rise, advances in early detection and treatment methods such as BRAF inhibitors and immune checkpoint inhibitors have led to a decline in mortality in recent years; however, acquired resistance remains a major challenge. USP28 exhibits a context-dependent dual role in melanoma: on one hand, high USP28 expression is associated with an immunosuppressive microenvironment (e.g., reduced NK cell infiltration), high tumor mutational burden (TMB), and microsatellite instability (MSI), potentially serving as a biomarker for predicting response to anti-CTLA-4 immunotherapy; on the other hand, in some cases, USP28 deficiency exhibits a pro-resistance effect—specifically, by stabilizing BRAF protein and abnormally activating the MAPK pathway, it induces tumor resistance to BRAF inhibitors (58). Vemurafenib and dabrafenib are selective BRAF V600E inhibitors that block MAPK/ERK signaling. Rigosertib interferes with RAS pathway signaling and is used to overcome BRAF inhibitor resistance (58). This mechanism suggests that low USP28 expression may be a negative predictive indicator for BRAF-targeted efficacy in these patients; for such scenarios, Rigosertib holds promise as an alternative treatment strategy to overcome resistance. In summary, the role of USP28 in tumors is not an absolute oncogenic switch but possesses tumor heterogeneity and microenvironment dependence. This suggests that in clinical trials, USP28 inhibition must be combined with companion diagnostics of the patient’s specific genetic mutation profile (e.g., BRAF status). USP28 exhibits a paradoxical dual role in melanoma: high expression predicts anti-CTLA-4 response in immune-infiltrated subsets, whereas its loss drives BRAF inhibitor resistance via MAPK hyperactivation—an example of genetic-context-dependent functional inversion.

### 3.12 Breast Cancer

Breast cancer is the most common malignant tumor in women globally, exhibiting high molecular heterogeneity. Among subtypes, Triple-Negative Breast Cancer (TNBC) lacks effective targets, with chemotherapy resistance and recurrence being the main difficulties in treatment; whereas hormone receptor-positive breast cancer often faces endocrine therapy resistance (87, 101). The role of USP28 in breast cancer (particularly invasive ductal carcinoma) presents complexity. On one hand, some studies indicate that USP28 exerts pro-carcinogenic effects in various cancers by stabilizing nuclear proteins such as c-MYC (29); on the other hand, evidence also suggests that in mouse models of breast cancer, USP28 deficiency paradoxically promotes malignant tumor progression, including epithelial-mesenchymal transition (EMT), proliferation, migration, and angiogenesis, an effect independent of HIF-1α, p53, and 53BP1 pathways (14).

This dual role suggests that the function of USP28 in breast cancer may be highly dependent on tumor subtype, microenvironment, or genetic background. In invasive ductal carcinoma, high USP28 expression is even positively correlated with improved patient survival rates, exhibiting tumor-suppressive characteristics (102). Therefore, targeting USP28 in breast cancer requires careful evaluation of its tissue-specific functions, and future research needs further subdivision to clarify its exact role to avoid potential adverse effects from blind inhibition. The tumor-suppressive role of USP28 in invasive ductal carcinoma, independent of p53 and HIF-1α pathways, contrasts sharply with its oncogenic functions in other solid tumors, suggesting engagement of distinct tissue-specific cofactors or competitive E3 ligases.

### 3.13 Determinants of USP28 functional duality: an integrated mechanistic perspective

The molecular basis of USP28 substrate selectivity and functional duality is governed by a multi-layered regulatory network rather than the catalytic domain alone.

First, tissue-specific cofactors and scaffolding proteins shape substrate availability. In hepatocellular carcinoma, downregulation of miR-216b enables preferential recruitment of KRT1 to sustain IFITM3 expression (42); in triple-negative breast cancer, USP28 stabilizes RecQ family helicases to support cell-cycle progression (59). The cellular context therefore determines which substrate is stabilized.

Second, oligomeric state transitions modulate substrate accessibility. Under basal conditions, USP28 forms a dimer that restricts recruitment of the PAF1c complex. Genotoxic stress disrupts the USP28-53BP1 interaction, promoting dimer dissociation and exposing interfaces for MYC stabilization and PAF1c recruitment (17).

Third, competitive E3 ligases influence USP28 activity. USP28 and FBXW7 exert antagonistic effects on shared substrates including c-MYC and NICD. In CLL with del(11q), heterozygous loss of USP28 paradoxically reduces NOTCH1 signaling, suggesting that the context of USP28 loss (hematologic vs. solid tumor) and the availability of alternative E3 ligases determine functional outcomes (60).

Fourth, microenvironmental signals redirect USP28 function. Hypoxia activates USP28 through SENP1-mediated deSUMOylation, leading to HIF-1α accumulation (20). ALKBH1-mediated m6A demethylation increases USP28 mRNA stability, establishing an ALKBH1-USP28-MYC axis that drives M2 macrophage polarization and suppresses CD8^+^ T-cell and natural killer cell infiltration, generating an immunosuppressive microenvironment (103). DNA damage alters the 53BP1-USP28 binding equilibrium, redirecting the enzyme from p53 stabilization toward MYC maintenance (17).

Fifth, genetic background determines functional outcomes. In BRAF-mutant melanoma, USP28 loss stabilizes BRAF and drives resistance to BRAF inhibitors (58); p53 mutation status dictates whether the USP28-53BP1 axis engages in DNA damage responses.

Finally, the immune context modifies USP28 relevance. Pan-cancer analyses associate USP28 expression with neutrophil and natural killer cell abundance. In melanoma, USP28 levels predict response to anti-CTLA-4 therapy (104); in hepatocellular carcinoma models, USP28 inhibition combined with anti-PD-1 therapy increases CD8^+^ T-cell infiltration (105).

Collectively, USP28 functional output is the integrated result of tissue-specific substrate availability, E3 competition, oligomeric state, microenvironmental signals, genetic background, and immune context—rather than a binary oncogene or tumor suppressor classification. These determinants operate concurrently to set the functional threshold of USP28 in a given tumor.

## 4 Clinical translation potential of USP28

Given the core role of USP28 in regulating the stability of key oncoproteins in various malignant tumors, its translational value as a clinical diagnostic marker, prognostic assessment indicator, and therapeutic target is becoming increasingly prominent. Current research is dedicated to translating basic research findings into clinical applications, primarily focusing on developing high-specificity small-molecule inhibitors, constructing USP28-based prognostic prediction models, and exploring combination application strategies for overcoming therapeutic resistance. The translational applications of USP28 as a biomarker and therapeutic target are outlined in **Table 2**.

**Table 2:** Translational Potential of USP28: From Biomarker Discovery to Therapeutic Strategies.

Application field	Disease/context	Biological type	Molecular mechanism/evidence	Reference
Biomarker - Diagnosis/Prognosis	Hepatocellular carcinoma (HCC)	Tissue protein/mRNA	USP28 upregulation is independent risk factor for shortened OS and DFS; multi-gene signature identifies high-risk metastasis patients	(42) (105)
	Non-small cell lung cancer (NSCLC)	Tissue protein/mRNA	USP28 upregulation serves as molecular indicator for poor survival prognosis	(48)
	Pancreatic cancer	Tissue protein/mRNA	USP28 upregulation is potential diagnostic biomarker for malignant progression and poor prognosis	(49)
	Bladder cancer	Tissue protein/mRNA	USP28/p53 co-expression pattern improves accuracy of tumor progression risk prediction	(56)
	Melanoma	Tissue protein/mRNA	USP28 expression level predicts CTLA-4 inhibitor response; USP28 deletion is negative biomarker for BRAF inhibitor efficacy	(58)
	Neuroblastoma	Tissue protein/mRNA	USP28 upregulation correlates with MYCN amplification and is poor prognostic biomarker for high-risk disease	(57)
	Pan-cancer	Tissue/multi-omics	USP28 expression correlates with immune infiltration (neutrophils, NK cells), MSI, and TMB	(104)
	Ovarian cancer	Cell/animal	USP28 inhibitor AZ1 disrupts USP28-SOX9 interaction, promotes SOX9 degradation, restores olaparib sensitivity	(25) (54)
Therapeutic Target - Small Molecule Inhibitors	Squamous cell carcinoma (SCC)	Cell/animal	Nanomolar AZ1 induces degradation of ΔNp63 and c-MYC, triggers apoptosis, induces tumor regression	(27) (28)
	Neuroblastoma	Cell/animal	AZ1 reduces MYCN stability and inhibits growth of high-risk neuroblastoma cells	(57)
	NSCLC/Esophageal cancer	Cell/animal	USP28 inhibition abrogates chemo/radioresistance and synergizes with cisplatin/radiotherapy	(47) (52)
	Lung squamous cell carcinoma	Cell/animal	USP28 inhibitor combined with statins produces synergistic antitumor effects	(26)
	CT1113 - R/R AML	Phase I clinical trial	First-in-human trial completed; excellent safety and PK profiles; preliminary efficacy observed; Phase II planned	(106)
	CT1113 - Cold tumors	Preclinical (animal)	Triggers CD8^+^ T-cell-dependent antitumor immunity; potentiates anti-PD-1 via YTHDF2 degradation; reverses immunosuppressive microenvironment	(41)
	CFTR (cystic fibrosis)	Cell-based	USP28-recruiting CFTR DUBTAC (MS9279) stabilizes ΔF508-CFTR mutant protein	(36)
Therapeutic Target - DUBTACs	cGAS-STING (cancer)	Cell-based	USP28-recruiting cGAS DUBTACs (MS2099/MS2100) stabilize cGAS, activate cGAS-STING signaling, suppress proliferation	(36)
	PPARγ (cancer metabolism)	Cell-based	First-in-class PPARγ DUBTACs (MS1727–MS1730) stabilize PPARγ; suppress cancer cell proliferation via metabolic reprogramming	(36)
	Ovarian cancer	Cell/animal	AZ1 + olaparib: reverses PARP inhibitor resistance	(25) (54)
	CLL	Primary cells	AZ1 + venetoclax: synergistic reduction in viability in high-NOTCH1 samples	(60)
Combination Strategies	Cold tumors	Animal	CT1113 + anti-PD-1: promotes CD8^+^ T-cell infiltration and enhances efficacy	(41)
	NSCLC	Cell/animal	USP28 inhibition + cisplatin/radiotherapy: overcomes chemo/radioresistance	(47) (52)
	Lung SCC	Cell/animal	USP28 inhibition + statins: synergistic antitumor effects	
				(26)



### 4.1 USP28 as a pan-cancer biomarker: integrating diagnosis, prognosis, and precision treatment decision-making

The clinical translational potential of USP28 as a biomarker spans three critical dimensions: tissue-based diagnosis/prognosis, non-invasive monitoring, and therapeutic decision-making.

First, in the realm of tissue diagnosis and prognostic stratification, accumulating evidence underscores a correlation between USP28 expression levels and tumor malignancy, staging, and patient survival outcomes across various cancer types. In solid tumors such as HCC, pancreatic cancer, NSCLC, and ovarian cancer, USP28 overexpression is a feature of malignant tissues and serves as an independent risk factor predictive of shortened OS and DFS (25, 42, 49, 54, 60, 107). Immunohistochemical analyses of clinical cohorts further reveal that co-expression patterns of USP28 with key oncoproteins, such as p53 and c-MYC, enhance the predictive accuracy for disease progression in bladder cancer and neuroblastoma (56, 57). Moreover, integrating bioinformatics with machine learning algorithms—specifically by incorporating USP28 into multi-gene signatures alongside ANGPT2 and DLL4—enables the identification of HCC subgroups at high risk of metastasis, thereby facilitating early intervention strategies (43). Nevertheless, the diagnostic utility of USP28 remains constrained by the absence of standardized expression thresholds to reliably distinguish malignant lesions from benign conditions.

Second, regarding non-invasive monitoring and prediction of therapeutic efficacy, USP28 exhibits promise. Its expression or activity status acts as an indicator for resistance to various targeted therapies and chemotherapeutic agents: it is associated with sorafenib and radiotherapy resistance in HCC (18, 102), osimertinib resistance in NSCLC (45, 47), and olaparib (PARP inhibitor) resistance in ovarian cancer (54). Of particular translational relevance is the observation that fluctuations in circulating levels of downstream effector molecules (e.g., phosphorylated STAT3 and stabilized c-MYC) hold potential as liquid biopsy markers for the real-time, non-invasive assessment of treatment response (108–110). Furthermore, pan-cancer analyses demonstrate a correlation between USP28 expression and immune microenvironment metrics, including tumor immune infiltration (neutrophils and natural killer cells), microsatellite instability (MSI), and tumor mutational burden (TMB), suggesting its viability as a cross-cancer immune prognostic biomarker (45, 104).

Finally, in guiding therapeutic decision-making, USP28 status offers bidirectional clinical value. A paradigmatic example is observed in melanoma, where USP28 loss leads to sustained MAPK pathway activation via BRAF protein stabilization, thereby conferring resistance to BRAF inhibitors; in such contexts, BRAF monotherapy should be avoided in favor of alternative regimens, such as rigosertib (15, 58). Similarly, in CLL and clear cell renal cell carcinoma (ccRCC), the activity status of USP28 or its regulatory axes (e.g., NOTCH1, c-Myc) can predict patient sensitivity to combination therapies involving USP28 inhibitors and BCL-2 inhibitors or other targeted agents (55, 60). Despite these findings, the routine clinical implementation of USP28 is currently hindered by a lack of standardized multivariate prognostic statistics, such as hazard ratios (HR) and 95% confidence intervals (CI).

### 4.2 Overcoming therapeutic resistance via USP28 inhibition: emerging drugs, rational combinations, and future directions

Targeting the deubiquitinating enzyme activity of USP28 has emerged as a strategy to overcome therapeutic resistance, with research and development efforts focusing on three core pillars: direct inhibition, synergistic combination, and precision-guided application.

In terms of direct inhibition and drug discovery, several highly selective small-molecule inhibitors have advanced to preclinical evaluation. The lead compound, AZ1—a nanomolar-potency specific inhibitor—effectively disrupts the interaction between USP28 and substrates such as SOX9 and c-MYC, inducing oncoprotein degradation and suppressing tumor growth in models of ovarian cancer, neuroblastoma, NSCLC, and CLL (22, 44, 54, 57, 60). Current developments include structurally optimized derivatives of vismodegib and the novel DUBTACs technology (111). Deubiquitinase-targeting chimeras (DUBTACs) recruit USP28 to specific substrates to remove degradative ubiquitin chains. The heterobifunctional molecule positions USP28 in proximity to the substrate, where it cleaves K48- or K63-linked polyubiquitin chains to block proteasomal degradation. This strategy achieves targeted protein stabilization, which is conceptually distinct from the degradation mechanism of PROTACs (45, 46). Two USP28-directed DUBTACs have been reported: a CFTR DUBTAC stabilizes the cystic fibrosis mutant ΔF508-CFTR, and a cGAS DUBTAC increases cGAS protein levels to amplify cGAS-STING signaling (45). DUBTACs offer the advantage of functional reprogramming rather than catalytic suppression, but current designs rely on non-covalent USP28 ligands with modest affinity, and the substrate scope is restricted (47).

Regarding combination therapies and resistance reversal, USP28 represents a potential sensitization target. Given that its primary mechanism of resistance involves the stabilization of key survival proteins, USP28 inhibition can yield synergistic effects when combined with various modalities: (1) Synergy with Targeted Therapies: In ovarian cancer, co-administration with PARP inhibitors (e.g., olaparib) reverses resistance mediated by the USP28-SOX9 axis (25, 54); in NSCLC, it potentiates osimertinib efficacy (45); and in CLL, combination with the BCL-2 inhibitor venetoclax demonstrates efficacy in samples exhibiting high NOTCH1 activity (60). (2) Sensitization to Radio- and Chemotherapy: In diverse malignancies, including lung and esophageal cancers, targeting USP28 disrupts radiation resistance and cisplatin tolerance mediated by the stabilization of STAT3, c-Myc/HIF-1α, or MAST1, thereby enhancing therapeutic outcomes (46, 52). (3) Novel Combinatorial Approaches: In lung squamous cell carcinoma, USP28 deficiency enhances sensitivity to statins, suggesting that a USP28 inhibitor plus statin regimen constitutes a synergistic strategy (22). (4) Immunotherapy Combinations: In preclinical models, the dual USP25/28 inhibitor CT1113 significantly potentiates anti-PD-1 therapy by promoting CD8^+^ T cell infiltration and attenuating M2-like macrophage polarization in immunologically “cold” tumors. Mechanistically, CT1113 acts through promoting YTHDF2 degradation and abolishing YTHDF2-mediated complement activation and immunosuppression, providing a rationale for clinical evaluation of USP28 inhibition combined with immune checkpoint blockade (41).

### 4.3 Barriers to clinical translation of USP28-targeted therapies

Despite the recent breakthrough of CT1113 completing a first-in-human phase I trial in relapsed/refractory acute myeloid leukemia (R/R AML) with excellent safety and pharmacokinetic profiles and preliminary efficacy observed, several substantial barriers impede broader clinical translation (106).

Isoform selectivity remains the foremost challenge. AZ1 exhibits low-nanomolar potency against USP28 but concurrently inhibits USP25 with comparable affinity (27). Vismodegib derivatives and FT206 also target the shared catalytic pocket (14). Recent structure-merging approaches have further expanded the chemical space of dual USP25/28 inhibitors (112). Structural studies reveal that AZ1, vismodegib, and FT206 all bind to the same hydrophobic cleft between the thumb and palm subdomains, highly conserved between USP28 and USP25 (~57% sequence identity), explaining the observed bispecificity. This lack of selectivity carries biological risk, as USP25 regulates TRAF-mediated inflammatory signaling and immune homeostasis.

A second concern is resistance mutations. A key glutamate residue at position 366 in USP28 (E373 in USP25) plays a central role in pocket stability and catalytic activity. Mutation of this single residue dramatically reduces inhibitor potency while paradoxically increasing catalytic activity, creating a “one-mutation-defeats-all” scenario (14). Pharmacokinetic limitations further hinder development. DUB inhibitors generally exhibit low oral bioavailability and metabolic instability. Prodrug strategies, such as esterification, have been explored to improve membrane permeability and oral bioavailability of DUB inhibitors (113). USP28 inhibitors targeting the dimerization interface tend to have high molecular weight and hydrophobicity, limiting solubility and tissue penetration.

Future strategies to address these barriers include: (1) structure-based design exploiting differences in dimerization interfaces to develop allosteric inhibitors unique to USP28; (2) prodrug strategies such as esterification to improve membrane permeability; (3) combination regimens—co-administration with anti-PD-1 (CT1113) (41), venetoclax (AZ1 in CLL) (60), or PARP inhibitors (AZ1 in ovarian cancer) (25, 54); and (4) exploration of USP28-based DUBTACs as an alternative therapeutic modality that enables targeted protein stabilization rather than catalytic suppression (36).

## 5 Summary and outlook

USP28 is a ubiquitin-specific protease with a constitutively active dimeric structure, whose functional involvement spans two major realms: maintenance of genomic stability and tumor development. The catalytic domain of this protein stabilizes key substrates through deubiquitination modifications; the C-terminal domain regulates the balance of DNA damage response pathways by mediating interactions with proteins such as 53BP1 and DTX3L. Under physiological conditions, USP28 stabilizes p53 by forming a complex with 53BP1 to activate the G1 checkpoint and synergizes with DTX3L to regulate the choice between non-homologous end joining (NHEJ) and homologous recombination (HR) repair, serving as a key hub for cells responding to genotoxic stress. When USP28 expression is abnormal, on one hand, it is highly expressed in most solid tumors such as lung cancer, pancreatic cancer, and ovarian cancer, driving cell proliferation, metabolic reprogramming, and therapeutic resistance by stabilizing oncoproteins like c-MYC, STAT3, SOX9, and FOXM1; on the other hand, its functional loss in specific contexts such as breast cancer may paradoxically promote epithelial-mesenchymal transition (EMT) and malignant progression, suggesting the complexity and environment-dependence of its function.

Currently, USP28-related research still faces numerous challenges. First, its tissue-specific regulatory mechanisms remain unclear, necessitating a systematic analysis of how promoter elements, epigenetic modifications, and microenvironmental signals determine its pro-carcinogenic or tumor-suppressive attributes. Second, the structural basis of substrate selectivity is controversial; the lack of high-resolution structural data leaves the molecular mechanism of how USP28 distinguishes between different substrates via non-catalytic domain allostery or dimerization state switching unelucidated. Third, the clinical translation process is limited by the lack of precise patient stratification criteria, and the development and safety evaluation of highly selective inhibitors are still in early stages.

Future research directions could focus on the following four aspects: First, utilize time-resolved structural biology techniques such as cryo-electron microscopy and X-ray free-electron lasers to dynamically capture the conformational changes and substrate recognition processes of USP28 under stress conditions, providing a structural blueprint for designing highly selective allosteric inhibitors. Second, employ super-resolution microscopy and spatial transcriptomics to map the nanoscale spatiotemporal distribution of USP28 and its substrates at DNA damage foci and within the tumor microenvironment, revealing the spatial logic of its regulation of repair pathway choice. Third, construct multi-omics integrated analysis models, combining ubiquitination proteomics with clinical big data, to identify key molecular features determining USP28 functional heterogeneity and establish precise prognostic prediction systems. Fourth, advance preclinical validation of combination therapy strategies, specifically exploring the synergistic effects of USP28 inhibitors with PARP inhibitors, chemotherapy drugs, or immunotherapies, and developing tumor microenvironment-responsive targeted delivery systems to overcome potential toxicity. In addition, advance the development of USP28-based DUBTACs as a complementary strategy to small-molecule inhibition. Unlike catalytic suppression, DUBTACs enable targeted protein stabilization by recruiting USP28 to specific substrates such as cGAS or PPARγ (36). Future work should focus on expanding the substrate scope to include classical tumor suppressors and evaluating *in vivo* pharmacokinetics and antitumor efficacy of lead DUBTAC compounds. The aforementioned research strategies are expected to promote the translation of USP28-related basic research into clinical applications for precision diagnosis and treatment, providing perspectives for analyzing DNA damage response networks and tumor resistance mechanisms, and may facilitate progress in overcoming tumor treatment bottlenecks and realizing individualized precision medicine.

## Glossary

ATM: Ataxia Telangiectasia Mutated
BCG: Bacillus Calmette-Guérin
CCA: Cholangiocarcinoma
CHK2: Checkpoint Kinase 2
c-MYC: Cellular Myelocytomatosis Oncogene
CTLA-4: Cytotoxic T-Lymphocyte Associated Protein 4
ctDNA: Circulating Tumor DNA
DALYs: Disability-Adjusted Life Years
DDR: DNA Damage Response
DSB: Double-Strand Break
DUBTAC: Deubiquitinase-Targeting Chimera
DYRK2: Dual Specificity Tyrosine Phosphorylation Regulated Kinase 2
EBV: Epstein-Barr Virus
EGFR-TKI: Epidermal Growth Factor Receptor Tyrosine Kinase Inhibitor
EMT: Epithelial-Mesenchymal Transition
ESCC: Esophageal Squamous Cell Carcinoma
FOXK1: Forkhead Box K1
FOXM1: Forkhead Box M1
FOXC1: Forkhead Box C1
HCC: Hepatocellular Carcinoma
HIF-1α: Hypoxia-Inducible Factor 1 Alpha
HMGCR: 3-Hydroxy-3-Methylglutaryl-CoA Reductase
HR: Homologous Recombination
IFITM3: Interferon-Induced Transmembrane Protein 3
IL-17: Interleukin-17
KRT1: Keratin 1
LSD1: Lysine-Specific Demethylase 1 (also known as KDM1A)
LUSC: Lung Squamous Cell Carcinoma
MDM2: Mouse Double Minute 2 Homolog
MSI: Microsatellite Instability
MVA: Mevalonate/Mevalonic acid
NHEJ: Non-Homologous End Joining
NK cell: Natural Killer cell
NSCLC: Non-Small Cell Lung Cancer
OS: Overall Survival
PARP: Poly (ADP-ribose) Polymerase
PAF1c: PAF1 Complex
PKM2: Pyruvate Kinase M2
PLK1: Polo-like Kinase 1
SCC: Squamous Cell Carcinoma
SENP1: SUMO/Sentrin Specific Peptidase 1
SIRT1: Sirtuin 1
SOX9: SRY-Box Transcription Factor 9
SREBP2: Sterol Regulatory Element-Binding Protein 2
STAT3: Signal Transducer and Activator of Transcription 3
SUMO: Small Ubiquitin-like Modifier
TCF7L2: Transcription Factor 7 Like 2
TMB: Tumor Mutational Burden
TNBC: Triple-Negative Breast Cancer
TPS: Targeted Protein Stabilization
USP: Ubiquitin-Specific Protease
USP28: Ubiquitin Specific Peptidase 28
Wnt: Wingless-type MMTV Integration Site Family
